# Chemotherapy for the initial treatment of metastatic prostate adenocarcinoma and neuroendocrine carcinoma at diagnosis: real world application and impact in the SEER database (2004 –2018)

**DOI:** 10.3389/fonc.2023.1165188

**Published:** 2023-06-09

**Authors:** Shihua Wang, Ming Yin, Peng Wang, Edmund Folefac, J. Paul Monk, Fred K. Tabung, Steven K. Clinton

**Affiliations:** ^1^The Ohio State University Comprehensive Cancer Center and Arthur G. James Cancer Hospital, Columbus, OH, United States; ^2^Division of Medical Oncology, Department of Internal Medicine, The Ohio State University College of Medicine, The Ohio State University, Columbus, OH, United States

**Keywords:** prostate cancer, chemotherapy, SEER, adenocarcinoma, neuroendocrine

## Abstract

**Background:**

Randomized controlled phase III trials have reported significant improvements in disease response and survival with the addition of chemotherapy to androgen deprivation therapy for men presenting with metastatic prostate cancer. We examined the implementation of such knowledge and its impact within the Surveillance, Epidemiology, and End Results (SEER) database.

**Method:**

The administration of chemotherapy for men with an initial presentation of metastatic prostate cancer from 2004 to 2018 in the SEER database and its association with survival outcomes was examined. Kaplan–Meier estimates were applied to compare survival curves. Cox proportion hazard survival models were used to analyze the association of chemotherapy and other variables with both cancer- specific and overall survival.

**Result:**

A total of 727,804 patients were identified with 99.9% presenting with adenocarcinoma and 0.1% with neuroendocrine histopathology. Chemotherapy as initial treatment for men with *de novo* distant metastatic adenocarcinoma increased from 5.8% during 2004–2013 to 21.4% during 2014–2018. Chemotherapy was associated with a poorer prognosis during 2004–2013 but was associated with improved cancer-specific (hazard ratio (HR) = 0.85, 95% confidence interval (CI): 0.78–0.93, p=0.0004) and overall survival (HR= 0.78, 95% CI: 0.71–0.85, p < 0.0001) during 2014–2018. The improved prognosis during 2014–2018 was observed in patients with visceral or bone metastasis and most impactful for patients aged 71–80 years. These findings were confirmed by subsequent propensity score matching analyses. Furthermore, chemotherapy was consistently provided to 54% of patients with neuroendocrine carcinoma at diagnosis from 2004 to 2018. Treatment was associated with improved cancer-specific survival (HR= 0.62, 95% CI: 0.45–0.87, p=0.0055) and overall survival (HR= 0.69, 95% CI: 0.51–0. 94, p=0.0176) during 2014–2018 but not significant in earlier years.

**Conclusion:**

Chemotherapy at initial diagnosis was increasingly employed in men with metastatic adenocarcinoma after 2014 and consistent with the evolution of National Comprehensive Cancer Network (NCCN) guidelines. Benefits for chemotherapy are suggested after 2014 in the treatment of men with metastatic adenocarcinoma. The use of chemotherapy for neuroendocrine carcinoma at diagnosis has remained stable, and outcomes have improved in more recent years. Further development and optimization of chemotherapy continues to evolve for men with *de novo* diagnosis of metastatic prostate cancer.

## Introduction

The burden of metastatic prostate cancer to society is enormous, both in terms of health care resources and human suffering; thus, the implementation of knowledge derived from quality clinical trials to community practice is imperative (1). Prostate cancer continues to be the most frequently diagnosed non-cutaneous cancer in American men and the second leading cause of cancer-related death (1), suggesting a critical need for improved screening and early diagnosis at a curable stage, and enhanced efficacy of therapy for advanced metastatic disease. Following the US Preventive Services Task Force (USPSTF) report in 2012 (2), there was a significantly reduced utilization of prostate cancer screening with prostate-specific antigen (PSA) testing, resulting in a lower overall detection of prostate cancer, but an unfortunate increase has emerged in the proportion of men presenting at advanced stages (1, 3–5). For example, recent data show a significant 41% increase in metastatic prostate cancer from 2010 to 2018 in men aged 45 –75 (3). This report focuses upon the treatments provided to the subgroup of men presenting with *de novo* metastatic disease in the real-world setting.

For decades, suppression of testosterone by castration or androgen deprivation therapy (ADT) has been the cornerstone of life-prolonging therapy for metastatic prostate cancer (6) and continues to improve with newer agents targeting specific components of the androgen signaling pathway (7–9). Yet, metastatic disease is essentially incurable, and mortality is nearly 70% by 5 years after diagnosis (10–13). Sadly, the median survival for men with castrate-resistant prostate cancer (CRPC) ranges from 18 to 24 months in most studies (12, 14, 15). Cytotoxic chemotherapy emerged as a beneficial treatment modality for metastatic CRPC, initially in the management of pain with mitoxantrone (16) and subsequently with docetaxel prolonging survival in landmark phase III trials by 2004 (17, 18) and supported by subsequent studies (19, 20). Soon thereafter, cabazitaxel, a second-generation taxane, showed a survival benefit in docetaxel refractory CRPC (21). With success in CRPC in the metastatic setting, the potential of adding taxane chemotherapy to androgen deprivation therapy (ADT) for men who present at initial diagnosis with treatment-naive metastatic disease was investigated in studies demonstrating improved overall survival and improved secondary endpoints such as prostate−specific antigen (PSA) failure and time to recurrence, particularly for those with higher volume disease (10, 11, 22, 23). National Comprehensive Cancer Network (NCCN) guidelines recommend ADT with docetaxel for six cycles as one of several options for the initial treatment of castration-naive metastatic prostate adenocarcinoma and was first included in the 2014 update (10, 24).

Our objective is to assess how the studies of chemotherapy combined with hormone therapy over recent decades have translated into real-world clinical practice for men with a new diagnosis of metastatic prostate cancer. The present study provides a comprehensive and contemporary (2004 –2018) summary of the large Surveillance, Epidemiology, and End Results (SEER) database. We also report the impact of initial chemotherapy on survival based upon the histopathological subtype and a number of relevant clinical and demographic factors.

## Methods

### Data source

We employed the population-based SEER Research Plus Data, 18 registries (2000–2018) using the SEERStat 8.3.9 software to identify patients 18 and older with an initial diagnosis of prostate cancer. We included those diagnosed during 2004–2018 because SEER collected PSA information since 2004 and the modern chemotherapy regimens (e.g., docetaxel) for metastatic disease were supported by clinical trial results in 2004. Those with stage Tis or T0 (no indication of cancer), with unknown T, N, and M stages and unknown survival time were excluded from the study. The primary endpoints were prostate-cancer-specific survival and overall survival. Based on the International Classification of Diseases for Oncology, Third Edition (ICD-O-3), we only included patients with prostate adenocarcinoma (8,140) or neuroendocrine carcinoma (8,012, 8,013, 8,041, 8,042, 8,045, 8,240, 8,241, 8,246, and 8,574) (25, 26). The SEER registries collect information on the first course of treatment. Chemotherapy data are categorized as either “yes— patient had chemotherapy” or “no/unknown— no evidence of chemotherapy was found in the medical records examined.” Patients with *de novo* distant metastatic disease were defined by M stage as 1. M stage was further grouped into M1a, M1b, M1c, and M1x. The following demographic and clinicopathological variables were included: age at diagnosis; PSA concentration; ethnicity (White, Black, Asian or Pacific Islander, and other); marital status; region of the US; Gleason score; T, N, and M stage; and treatments including surgery, radiotherapy, and chemotherapy. As the data are de-identified, institutional review board approval was not necessary for this project.

### Statistical analysis

Continuous data were evaluated by T-test. Square root or log transformation of the original data was applied to satisfy the assumption of equal variances. Categorical data were compared using the Pearson’s chi‐square test. The trend for the proportion of patients receiving chemotherapy was examined by the Cochran–Armitage test. Survival curves were defined by Kaplan–Meier methodology and compared through log rank testing. Univariate and multivariate Cox proportional hazards regression analyses were utilized to examine the impact of chemotherapy and predictors on cancer- specific and overall survival. The multivariable model was constructed with a backward selection strategy with an entry level of 0.05 at every step. Only variables with a *p-*value < 0.10 in the univariate analyses were included, except that chemotherapy was always included in the multivariate analysis. To address potential disparities between patients treated with or without chemotherapy, impacts of chemotherapy on prognosis were determined in propensity score matching analyses. Matching variables included age; PSA; Gleason score; T, N, and M stages; race; marital status; region; and local treatment. All statistical tests were two-sided with *p*< 0.05 to be significant. Data analyses were performed using SAS 9.4 (Raleigh, NC).

## Results

### Patients with prostate adenocarcinoma or neuroendocrine carcinoma diagnosed during 2004–2018

A total of 727,804 patients diagnosed with prostate cancer during 2004–2018 (**Supplementary Table S1**) were identified with 727,133 (99.9%) having adenocarcinoma and 671 (0.1%) with neuroendocrine histopathology. Those with neuroendocrine cancer at presentation were older and exhibited higher PSA and greater Gleason grade. The proportion of men with metastatic adenocarcinoma at diagnosis was 3%, which was much lower than 57% of those with neuroendocrine histology (**Supplementary Table S1**).

### Time trends for chemotherapy administration for metastatic prostate cancer at diagnosis during 2004–2018

**Figure 1:**
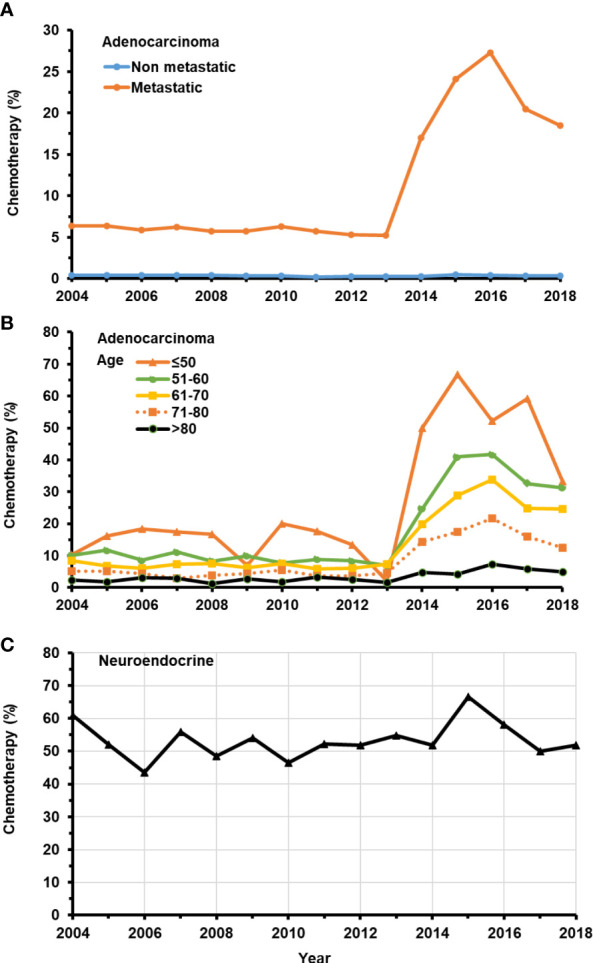
Temporal change in the percentage of patients with *de novo* metastatic prostate adenocarcinoma receiving chemotherapy from 2004–2018. **(A)** Patients with or without *de novo* metastatic prostate adenocarcinoma. **(B)** Patients in different age groups with *de novo* metastatic prostate adenocarcinoma. **(C)** All patients with prostate neuroendocrine carcinoma.

As expected, the proportion of men with non-metastatic adenocarcinoma receiving chemotherapy was between 0.2% and 0.5% over time (*p* trend <0.0001) (**Figure 1A**). For men with metastatic adenocarcinoma at presentation, chemotherapy was provided to 5.8% during years 2004–2013 and increased to 21.4% during the years of 2014–2018 (*p* trend < 0.0001) (**Figure 1A**). In this population, the administration of chemotherapy was strongly age dependent after 2013 (all *p* trends < 0.0001) (**Figure 1B**; **Table 1**), with younger men more likely to receive chemotherapy. A much high proportion of men presenting with neuroendocrine cancer received chemotherapy (54%), and the proportion remained steady between 2004 and 2018, with year-to-year variation due to the overall smaller number of cases compared to adenocarcinoma (*p* trend=0.1349) (**Figure 1C**).

**Table 1:** Descriptive characteristics of patients with a *de novo* diagnosis of metastatic prostate adenocarcinoma who were initially treated with or without chemotherapy during 2004–2013 and 2014–2018.

Variable	2003–2014 n (%)			2014–2018 n (%)		
	No Chemotherapy 12,451 (94.2)	Chemotherapy 772 (5.8)	*p*-value	No Chemotherapy 8,471 (78.6)	Chemotherapy 2,310 (21.4)	*p*-value
Age (years)						
Mean ± SD	70.3 ± 10.8	64.9 ± 10.1	<0.0001	71.7 ± 10.1	65.1 ± 8.9	<0.0001
Median (range)	70 (35–100)	64 (38–98)		72 (39–100)	65 (34–95)	
Distribution			<0.0001			<0.0001
≤50	368 (3).	60 (8)		119 (1)	134 (6)	
51–60	2,132 (17)	211 (27)		1,088 (13)	564 (24)	
61–70	3,788 (30)	278 (36)		2,754 (33)	990 (43)	
71–80	3,650 (29)	164 (21)		2,645 (31)	515 (22)	
>80	2,513 (20)	59 (8)		1,865 (22)	107 (5)	
PSA (ng/ml)						
Mean ± SD	62.7 ± 38.1	62.2 ± 39.6	0.6884	61.3 ± 38.0	66.2 ± 37.0	<0.0001
Median (range)	83.7 (0.1–99.8)	88 (0.1–99.8)		73.2 (0.1–99.8)	98 (0.1–99.8)	
Distribution			0.0044			<0.0001
<20.0	2,761 (22)	205 (27)		2,008 (24)	448 (19)	
20–90.0	3,239 (26)	164 (21)		2,347 (28)	628 (27)	
>90	5,691 (46)	360 (47)		3,740 (44)	1,178 (51)	
Unknown	760 (6)	43 (6)		376 (4)	56 (2)	
Gleason score			<0.0001			<0.0001
≤6	560 (5)	22 (2.9)		129 (2)	23 (1)	
7	2,117 (17)	101 (13)		1,072 (13)	172 (8)	
8	2,629 (21)	132 (17)		1,819 (21)	415 (18)	
9–10	5,355 (43)	403 (52)		4,185 (49)	1,425 (62)	
Unknown	1,790 (14)	114 (15)		1,266 (15)	275 (12)	
T stage			<0.0001			0.0066
T1	3,977 (32)	221 (29)		2,786 (33)	728 (32)	
T2	5,056 (41)	273 (35)		2,934 (35)	779 (34)	
T3	1,512 (12)	114 (15)		1,433 (17)	374 (16)	
T4	1,906 (15)	164 (21)		1,318 (16)	429 (19)	
N stage			<0.0001			<0.0001
N0	9,035 (73)	487 (63)		5,316 (63)	1,145 (50)	
N1	3,416 (27)	285 (37)		3155 (37)	1,165 (50)	
M stage			<0.0001			<0.0001
M1a	771 (6)	53 (7)		739 (9)	126 (5)	
M1b	8,914 (72)	480 (62)		6,013 (71)	1,607 (70)	
M1c	2,403 (19)	202 (26)		1,080 (13)	425 (18)	
M1x	363 (3)	37 (5)		639 (7)	152 (7)	
Marital status			<0.0001			0.0002
Married	7,188 (58)	517 (67)		4,885 (58)	1,442 (62)	
Unmarried^#^	4,390 (35)	208 (27)		3,030 (36)	732 (32)	
Unknown	873 (7)	47 (6)		556 (7)	136 (6)	
Race			0.0339			0.1817
White	9,185 (74)	599 (78)		6,368 (75)	1,769 (77)	
Black	2,473 (20)	138 (18)		1,459 (17)	387 (17)	
Other	739 (6)	35 (5)		567 (7)	142 (6)	
Unknown	54 (0.4)	0 (0)		77 (1)	12 (1)	
Region			0.1650			0.5063
West	6,140 (49)	352 (45.6)		4,446 (53)	1,188 (51)	
South	3,007 (24)	191 (24.7)		2,037 (24)	558 (24)	
Midwest	1,415 (11)	102 (13.2)		859 (10)	229 (10)	
Northeast	1,889 (15)	127 (16.5)		1,129 (13)	335 (15)	
Local treatment			<0.0001			<0.0001
No local treatment	8,144 (65)	411 (53)		5,359 (63)	1,613 (70)	
Radiotherapy only	2,578 (21)	250 (32)		1,746 (21)	468 (20)	
Surgery only	1,435 (12)	73 (10)		1,107 (13)	178 (8)	
Radiotherapy and surgery	294 (2)	38 (5)		259 (3)	51 (2)	

# Unmarried including divorced, separated, single (never married), unmarried or domestic Partner, widowed.

### Characteristics of patients with metastatic prostate adenocarcinoma at diagnosis and initially treated with or without chemotherapy during 2004–2013 and 2014–2018

**Table 1** outlines factors contributing to the selection of chemotherapy for initial treatment of men presenting with metastatic prostate adenocarcinoma at primary diagnosis for the intervals of 2004–2013 (5.8% receiving chemotherapy) and 2014–2018 (21.4% receiving chemotherapy). Younger age at diagnosis was strongly associated with selection of initial chemotherapy particularly after 2013 (**Table 1**). Patients with cancers characterized by higher Gleason score (9, 10), more advanced T stage, positive lymph node metastasis, and more advanced M stage (M1c) were significantly more likely to receive chemotherapy in both periods. A higher PSA (> 90 ng/ml) emerged as a modest predictor for chemotherapy treatment during 2014–2018.

### Impact of chemotherapy on cancer- specific and overall survival in patients presenting with metastatic prostate adenocarcinoma during 2004–2013 and 2014–2018

**Figure 2:**
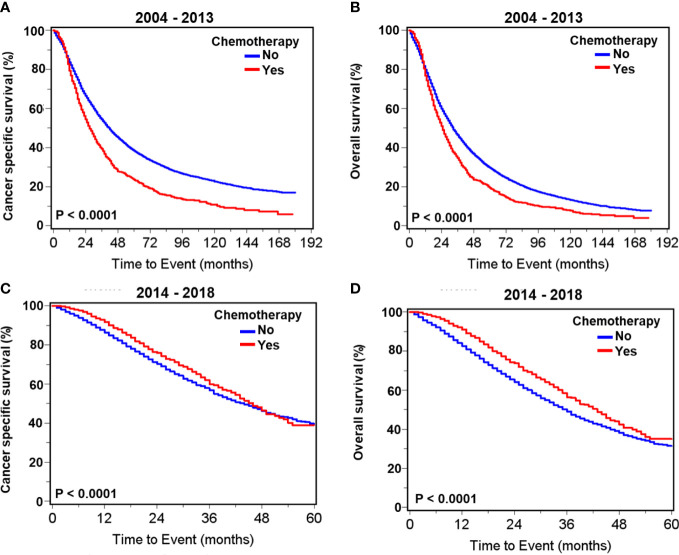
Kaplan–Meier survival curves for cancer- specific and overall survival in all patients with *de novo* metastatic prostate adenocarcinoma with or without chemotherapy. For patients diagnosed during 2004–2013, curves of cancer- specific survival **(A)** and overall survival **(B)**. For patients diagnosed during 2014–2018, curves of cancer- specific survival **(C)** and overall survival **(D)**.

During 2004–2013, there were significantly higher proportions of both cancer-specific and overall deaths in metastatic patients receiving chemotherapy compared with those receiving no chemotherapy (**Supplementary Table S2**; **Figure 2**). In contrast, during 2014–2018, the proportion of overall death in patients receiving chemotherapy was significantly less than in those without chemotherapy, while cancer-specific death was not significantly impacted by chemotherapy selection (**Supplementary Table S2**). Survival curves illustrate that chemotherapy was associated with significantly worse cancer-specific and overall survival in patients with metastatic prostate adenocarcinoma carcinoma during 2004–2013 (**Figures 2A, B**) but was associated with significantly improved prognoses during 2014–2018 (**Figures 2C, D**).

**Table 2** presents multivariate survival analyses showing greater depth of insight with reduced bias. Chemotherapy was associated with significantly improved cancer-specific survival (HR= 0.85, 95% CI: 0.78 –0.93, p=0.0004) and overall survival (HR= 0.78, 95% CI: 0.71 –0.85, *p*<0.0001) in patients with metastatic prostate adenocarcinoma diagnosed during 2014–2018. In comparison, during the period of 2004–2013, chemotherapy was associated with significantly worse cancer-specific survival (HR =1.48, 95% CI: 1.37 –1.61, *p*<0.0001) and overall survival (HR =1.39, 95% CI: 1.28 –1.50, *p*<0.0001) (**Table 2**). Other factors significantly predicting poor outcomes in both time intervals were greater age, higher PSA, T4 stage, extensive metastasis beyond M1a, and higher Gleason score. The inclusion of radiotherapy or surgery to the initial treatment plan was not associated with a change in cancer-specific or overall survival during either time period. However, combined radiotherapy and surgery was associated with significantly worse survival during the earlier time frame of 2004–2013 (**Table 2**). Men who were married, as an indicator of support systems, fared significantly better than those who were not by 18% –25%. Men with metastatic prostate cancer living in the South fared significantly worse than in other regions regardless of treatment and time interval.

**Table 2:** Multivariate survival analyses of variables associated with survival in patients with metastatic prostate adenocarcinoma diagnosed during 2004–2013 and 2014–2018.

Variable	Cancer-specific survival				Overall survival			
	2004–2013		2014–2018		2004–2013		2014–2018	
	HR (95% CI)	*p*-value	HR (95% CI)	*p*-value	HR (95% CI)	*p*-value	HR (95% CI)	*p*-value
Age (years)								
≤50	1		1		1		1	
51–60	0.96 (0.85–1.08)	0.5143	0.83 (0.65–1.06)	0.1337	1.00 (0.89–1.12)	0.9889	0.82 (0.65–1.03)	0.0806
61–70	0.96 (0.86–1.08)	0.5232	0.91 (0.72–1.15)	0.4317	1.08 (0.96–1.21)	0.1879	0.96 (0.77–1.19)	0.6824
71–80	1.13 (1.01–1.28)	0.0387	1.15 (0.91–1.45)	0.2492	1.37 (1.23–1.53)	<0.0001	1.22 (0.98–1.52)	0.0755
>80	1.52 (1.35–1.72)	<0.0001	1.56 (1.23–1.98)	0.0003	2.03 (1.81–2.27)	<0.0001	1.74 (1.39–2.17)	<0.0001
PSA (ng/ml)								
<20.0	1		1		1		1	
20–90.0	1.28 (1.2–1.36)	<0.0001	1.23 (1.1–1.37)	0.0002	1.22 (1.16–1.29)	<0.0001	1.22 (1.11–1.35)	<0.0001
>90	1.58 (1.49–1.68)	<0.0001	1.51 (1.36–1.66)	<0.0001	1.49 (1.41–1.57)	<0.0001	1.46 (1.34–1.6)	<0.0001
Unknown	1.37 (1.24–1.51)	<0.0001	1.68 (1.39–2.04)	<0.0001	1.37 (1.26–1.50)	<0.0001	1.70 (1.43–2.01)	<0.0001
T stage								
T1	1.06 (0.98–1.14)	0.1547	1.34 (1.19–1.51)	<.0001	1.07 (1.00–1.14)	0.0561	1.34 (1.21–1.50)	<0.0001
T2	1.06 (0.98–1.13)	0.1283	1.31 (1.16–1.47)	<.0001	1.07 (1.01–1.14)	0.0248	1.28 (1.15–1.42)	<0.0001
T3	1		1		1		1	
T4	1.43 (1.32–1.55)	<0.0001	1.77 (1.56–2.01)	<0.0001	1.39 (1.30–1.50)	<0.0001	1.71 (1.52–1.91)	<0.0001
N stage								
N0	1				1			
N1	1.10 (1.05–1.16)	0.0002			1.07 (1.02–1.11)	0.0060		
M stage								
M1a	1		1		1		1	
M1b	1.60 (1.45–1.77)	<0.0001	1.73 (1.47–2.04)	<.0001	1.38 (1.27–1.50)	<0.0001	1.66 (1.43–1.91)	<0.0001
M1c	1.95 (1.75–2.17)	<0.0001	2.30 (1.92–2.75)	<.0001	1.64 (1.50–1.80)	<0.0001	2.11 (1.80–2.47)	<0.0001
M1x	1.62 (1.38–1.89)	<0.0001	1.81 (1.47–2.23)	<.0001	1.44 (1.26–1.64)	<0.0001	1.83 (1.52–2.2)	<0.0001
Gleason score								
≤6	0.68 (0.59–0.79)	<0.0001	0.78 (0.52–1.19)	0.2477	0.80 (0.71–0.89)	<.0001	0.79 (0.55–1.13)	0.2028
7	1				1		1	
8	1.23 (1.14–1.32)	<0.0001	1.14 (0.97–1.33)	0.1089	1.13 (1.06–1.20)	0.0002	1.13 (0.98–1.30)	0.0842
9–10	1.79 (1.68–1.91)	<0.0001	1.98 (1.72–2.27)	<0.0001	1.60 (1.51–1.69)	<0.0001	1.84 (1.63–2.07)	<0.0001
Unknown	1.63 (1.50–1.77)	<0.0001	2.32 (1.98–2.72)	<0.0001	1.47 (1.37–1.57)	<0.0001	2.16 (1.88–2.48)	<0.0001
Local treatment								
No treatment			1					
Radiotherapy only			1.04 (0.96–1.14)	0.3435				
Surgery only			1.02 (0.91–1.14)	0.7578				
Radiotherapy and surgery			1.33 (1.08–1.63)	0.0066				
Chemotherapy								
No	1		1		1		1	
Yes	1.48 (1.37–1.61)	<0.0001	0.85 (0.78–0.93)	0.0004	1.39 (1.28–1.50)	<0.0001	0.78 (0.71–0.85)	<0.0001
Marital status								
Married	1		1		1		1	
Unmarried#	1.18 (1.13–1.24)	<0.0001	1.18 (1.10–1.28)	<0.0001	1.22 (1.17–1.27)	<0.0001	1.25 (1.17–1.34)	<0.0001
Unknown	0.95 (0.87–1.04)	0.2353	0.88 (0.75–1.03)	0.1073	0.96 (0.89–1.04)	0.3059	0.92 (0.80–1.06)	0.2490
Race								
White	1		1		1		1	
Black	0.98 (0.92–1.03)	0.3815	0.94 (0.85–1.04)	0.236	0.98 (0.94–1.04)	0.5274	0.94 (0.86–1.03)	0.1606
Other	0.74 (0.67–0.82)	<0.0001	0.64 (0.54–0.76)	<0.0001	0.79 (0.72–0.86)	<0.0001	0.69 (0.59–0.80)	<0.0001
Unknown	0.26 (0.14–0.47)	<0.0001	0.19 (0.07–0.50)	0.0009	0.43 (0.29–0.64)	<0.0001	0.23 (0.10–0.50)	0.0003
Region								
West	1		1		1		1	
South	1.16 (1.10–1.23)	<0.0001	1.14 (1.05–1.25)	0.0032	1.18 (1.12–1.24)	<.0001	1.21 (1.12–1.31)	<0.0001
Midwest	1.06 (0.99–1.14)	0.1171	1.11 (0.99–1.25)	0.0833	1.10 (1.03–1.17)	0.0029	1.17 (1.05–1.30)	0.0038
Northeast	1.00 (0.94–1.07)	0.9967	0.94 (0.84–1.05)	0.2603	1.07 (1.01–1.13)	0.0162	1.02 (0.92–1.12)	0.7366

^#^Unmarried including divorced, separated, single (never married), unmarried or domestic partner, and widowed.

Age at diagnosis, which clearly impacted selection of chemotherapy, appears to be associated with survival response to chemotherapy. We observed no significant improvement in overall or cancer-specific survival for younger men <70 years or for those over 80. In contrast, improved cancer- specific and overall survival were seen among men aged 71–75 and 76–80 years (**Table 3**).

**Table 3:** Multivariate survival analyses of impacts of chemotherapy on survival in patients with *de novo* metastatic prostate adenocarcinoma among different age groups during 2014–2018.

Variable	Cancer- specific survival		Overall survival	
	HR (95% CI)	*p*-value	HR (95% CI)	*p*-value
Age groups				
≤ 70 years				
Chemotherapy (yes vs no)	1.00 (0.89–1.12)	0.9970	0.93 (0.84–1.03)	0.1712
71–75 years				
Chemotherapy (yes vs no)	0.75 (0.6–0.94)	0.0134	0.68 (0.55–0.85)	0.0005
76–80 years				
Chemotherapy (yes vs no)	0.65 (0.48–0.88)	0.0060	0.60 (0.45–0.79)	0.0004
> 80 years				
Chemotherapy (yes vs no)	1.01 (0.74–1.37)	0.9734	0.85 (0.64–1.13)	0.2656

The benefits of chemotherapy are related to metastatic disease burden at diagnosis. Our data showed that chemotherapy was associated with significantly improved cancer- specific and overall survival in patients with metastasis to either visceral (liver, lung, or brain), or bone alone. In contrast, chemotherapy was not associated with improvements in cancer-specific survival or overall survival in patients presenting with only distant lymph node metastasis (**Table 4**).

**Table 4:** Multivariate survival analyses of impacts chemotherapy on survival in patients with *de novo* metastatic prostate adenocarcinoma at varied metastatic sites during 2014–2018.

Metastatic site	Cancer- specific survival		Overall survival	
	HR (95% CI)	*p*-value	HR (95% CI)	*p*-value
Distant lymph node metastasis only				
Chemotherapy (yes vs no)	1.01 (0.61–1.66)	0.9741	0.79 (0.49–1.28)	0.3376
Bone metastasis only with or without lymph node metastasis				
Chemotherapy (yes vs no)	0.87 (0.78–0.96)	0.0059	0.59 (0.48–0.73)	<0.0001
Visceral metastasis (lung, liver or brain (with or without bone or lymph node metastasis)				
Chemotherapy (yes vs no)	0.63 (0.50–0.79)	<0.0001	0.58 (0.47–0.72)	<0.0001

### Propensity score matching analyses of impact of chemotherapy on prognosis in patients presenting with metastatic prostate adenocarcinoma during 2004–2013 and 2014–2018

After the propensity score matching, equal numbers of patients with comparable features treated with or without chemotherapy were selected in those having metastatic adenocarcinoma during 2004–2013 and 2014–2018 (**Supplementary Table S3**). We observed similar and consistent patterns of death and survival curves with propensity score matching. (**Supplementary Table S4**; **Figures S1A–D**). Most importantly, we confirmed that in patients with metastatic prostate adenocarcinoma, chemotherapy was associated with worse prognoses during 2004–2013 but improved cancer-specific and overall survival during 2014–2018 (**Table 5**).

**Table 5:** Multivariate analyses of risk factors correlated with survival in propensity score matched patients with metastatic prostate adenocarcinoma diagnosed during 2004–2013 and 2014–2018.

Variable	Cancer- specific survival				Overall survival			
	2004–2013		2014–2018		2004–2013		2014–2018	
	HR (95% CI)	*p*-value	HR (95% CI)	*p*-value	HR (95% CI)	*p*-value	HR (95% CI)	*p*-value
Age (years)								
≤50	1		1		1		1	
51–60	0.98 (0.78–1.24)	0.8659	0.84 (0.65–1.08)	0.1635	1.02 (0.82–1.28)	0.8346	0.81 (0.64–1.03)	0.0889
61–70	0.98 (0.78–1.23)	0.8723	0.94 (0.74–1.19)	0.5914	1.07 (0.86–1.33)	0.5353	1.00 (0.80–1.26)	0.9904
71–80	1.18 (0.93–1.50)	0.186	1.02 (0.79–1.31)	0.8895	1.36 (1.08–1.71)	0.0085	1.10 (0.87–1.40)	0.4122
>80	1.68 (1.25–2.27)	0.0006	1.52 (1.11–2.08)	0.0088	2.07 (1.57–2.71)	<0.0001	1.55 (1.15–2.08)	0.0036
PSA (ng/ml)								
<20.0	1		1		1		1	
20–90.0	1.19 (1.00–1.42)	0.0508	1.13 (0.96–1.34)	0.1468	1.12 (0.95–1.32)	0.1685	1.15 (0.98–1.34)	0.0931
>90	1.44 (1.24–1.67)	<0.0001	1.33 (1.14–1.55)	0.0003	1.36 (1.19–1.56)	<0.0001	1.36 (1.17–1.57)	<0.0001
Unknown	0.93 (0.70–1.26)	0.6520	1.38 (0.93–2.06)	0.1094	0.99 (0.76–1.29)	0.9427	1.57 (1.11–2.23)	0.0108
T stage								
T1	1		1		1		1	
T2	0.90 (0.77–1.04)	0.1576	0.99 (0.86–1.13)	0.8491	0.94 (0.82–1.08)	0.3940	1.00 (0.88–1.13)	0.9927
T3	0.85 (0.70–1.03)	0.1009	0.77 (0.65–0.93)	0.0054	0.86 (0.72–1.04)	0.1121	0.80 (0.68–0.94)	0.0082
T4	1.35 (1.14–1.60)	0.0005	1.23 (1.05–1.43)	0.0094	1.32 (1.13–1.55)	0.0006	1.25 (1.08–1.44)	0.0025
M stage								
M1a	1		1		1		1	
M1b	1.66 (1.28–2.16)	0.0001	1.75 (1.31–2.36)	0.0002	1.58 (1.25–2.00)	0.0002	1.69 (1.29–2.22)	0.0001
M1c	2.02 (1.53–2.65)	<0.0001	2.39 (1.75–3.26)	<0.0001	1.90 (1.48–2.44)	<0.0001	2.27 (1.71–3.02)	<0.0001
M1x	1.32 (0.89–1.94)	0.1648	1.83 (1.26–2.66)	0.0015	1.41 (1.00–1.98)	0.0534	1.98 (1.41–2.77)	<0.0001
Gleason score								
≤6	0.62 (0.38–1.02)	0.0576	1.25 (0.66–2.37)	0.4953	0.81 (0.54–1.21)	0.3003	1.03 (0.55–1.95)	0.9193
7	1		1		1		1	
8	1.07 (0.85–1.34)	0.5692	1.13 (0.85–1.50)	0.3998	1.03 (0.83–1.27)	0.8039	1.12 (0.86–1.44)	0.4043
9–10	1.52 (1.25–1.84)	<0.0001	2.12 (1.65–2.72)	<0.0001	1.43 (1.20–1.70)	<0.0001	2.01 (1.60–2.53)	<0.0001
Unknown	1.40 (1.11–1.77)	0.005	2.22 (1.66–2.97)	<0.0001	1.35 (1.09–1.67)	0.0065	2.21 (1.70–2.88)	<0.0001
Local treatment								
Nol treatment			1				1	
Radiotherapy only			1.09 (0.96–1.25)	0.1882			1.06 (0.94–1.20)	0.3266
Surgery only			0.89 (0.71–1.11)	0.3077			0.93 (0.75–1.14)	0.4543
Radiotherapy and surgery			1.65 (1.19–2.28)	0.0024			1.45 (1.06–1.99)	0.0200
Chemotherapy								
No	1		1		1		1	
Yes	1.57 (1.39–1.77)	<0.0001	0.85 (0.76–0.95)	0.0030	1.48 (1.33–1.65)	<0.0001	0.78 (0.70–0.86)	<0.0001
Marital status								
Married					1			
Unmarried#					1.21 (1.07–1.37)	0.0028		
Unknown					1.04 (0.82–1.33)	0.7425		
Race								
White	0.89 (0.76–1.04)	0.1372	1				1	
Black	1		1.02 (0.88–1.18)	0.8188			1.04 (0.91–1.20)	0.5424
Other	0.70 (0.49–1.00)	0.0473	0.62 (0.48–0.82)	0.0007			0.66 (0.52–0.85)	0.0013
Unknown			0.36 (0.09–1.44)	0.1483			0.31 (0.08–1.26)	0.1015
Region								
West	1				1		1	
South	1.20 (1.03–1.40)	0.0185			1.27 (1.11–1.45)	0.0007	1.14 (1.00–1.29)	0.0448
Midwest	1.13 (0.94–1.36)	0.1823			1.18 (1.00–1.40)	0.052	0.98 (0.82–1.16)	0.7858
Northeast	0.94 (0.79–1.13)	0.5243			1.05 (0.90–1.24)	0.5217	1.07 (0.92–1.25)	0.3815

# Unmarried including divorced, separated, single (never married), unmarried or domestic partner, and widowed.

### Baseline characteristics of patients with prostate neuroendocrine carcinoma with or without chemotherapy during 2004–2013 and 2014–2018

Younger patients with neuroendocrine carcinoma with lower serum PSA levels, more advanced T stage, lymph node and distant metastasis, and radiotherapy were more likely to receive chemotherapy during both periods. During 2004–2013, patents of 51–60 years old compared to older individuals were more likely to receive chemotherapy. In addition, during 2014–2018, patients of 61–70 years and from West and Northeast regions were more likely to receive chemotherapy (**Table 6**). Detailed treatments provided to these patients is presented in **Supplementary Table S5**.

**Table 6:** Descriptive characteristics of patients with a *de novo* diagnosis of prostate neuroendocrine carcinoma who were initially treated with or without chemotherapy during 2004–2013 and 2014–2018.

Variable	2004–2013 n (%)			2014–2018 n (%)		
	No chemotherapy 177 (48)	Chemotherapy 192 (52)	*p*-value	No chemotherapy 133 (44)	Chemotherapy 169 (56)	*p*-value
Age (years)						
Mean ± SD	72.8 ± 11.1	67.9 ± 11.3	<0.0001	73.3 ± 11.2	67.2 ± 9.3	<0.0001
Median (range)	73 (44–96)	68 (30–92)		73 (44–96)	67 (39–92)	
Distribution						
≤50	3 (2)	11 (6)	0.0009	3 (2)	10 (6)	<0.0001
51–60	21 (12)	41 (21)		16 (12)	22 (13)	
61–70	55 (31)	61 (32)		37 (28)	75 (44)	
71–80	46 (26)	51 (27)		38 (29)	51 (30)	
>80	52 (29)	28 (15)		39 (29)	11 (7)	
PSA (ng/ml)						
Mean ± SD	34.7 ± 39.7	22 ± 33.9	0.0010	35.7 ± 39.3	27.3 ± 36.8	0.0787
Median (range)	10.5 (0.1–99.8)	5.8 (0.1–99.8)		13.6 (0.1–99.8)	6.1 (0.1–99.8)	
Distribution			0.0422			0.0113
< 20.0	91 (51)	121 (63)		64 (48)	105 (62)	
20.0–90.0	23 (13)	16 (8)		18 (14)	24 (14)	
>90.0	36 (20)	23 (12)		25 (19)	27 (16)	
Unknown	27 (15)	32 (17)		26 (20)	13 (8)	
T stage			0.0002			0.0530
T1	36 (20)	30 (16)		27 (20)	20 (12)	
T2	69 (39)	41 (21)		36 (27)	38 (22)	
T3	18 (10)	36 (19)		23 (17)	27 (16)	
T4	54 (31)	85 (44)		47 (35)	84 (50)	
N stage			0.0003			0.0016
N0	118 (67)	92 (48)		77 (58)	67 (40)	
N1	59 (33)	100 (52)		56 (42)	102 (60)	
M stage			0.0006			0.0002
M0	102 (58)	74 (39)		68 (51)	44 (26)	
M1a	9 (5)	11 (6)		8 (6)	11 (7)	
M1b	27 (15)	36 (19)		21 (16)	41 (24)	
M1c	39 (22)	62 (32)		35 (26)	65 (38)	
M1X	0 (0)	9 (5)		1 (1)	8 (5)	
Gleason score			0.2540			0.0162
≤6	7 (4)	11 (6)		2 (2)	0 (0)	
7	8 (5)	3 (2)		10 (8)	5 (3)	
8	11 (6)	8 (4)		10 (8)	8 (5)	
9–10	53 (30)	50 (26)		45 (34)	43 (25)	
Unknown	98 (55)	120 (63)		66 (50)	113 (67)	
Local treatment						
No	76 (43)	65 (34)	<0.0001	66 (50)	70 (41)	0.0013
Radiotherapy only	24 (14)	65 (34)		18 (14)	53 (31)	
Surgery only	66 (37)	34 (18)		37 (28)	28 (17)	
Radiotherapy and surgery	11 (6)	28 (15)		12 (9)	18 (11)	
Marital status						
Married	109 (62)	139 (72)	0.0546	83 (62)	126 (75)	0.0510
Unmarried^#^	56 (32)	47 (24)		44 (33)	35 (21)	
Unknown	12 (7)	6 (3)		6 (5)	8 (5)	
Race						
White	150 (85)	175 (91)	0.1040	106 (80)	138 (82)	0.9540
Black	16 (9)	12 (6)		16 (12)	17 (10)	
Other	11 (6)	4 (2)		10 (8)	13 (8)	
Unknown	0 (0)	1 (1)		1 (1)	1 (1)	
Region						
West	96 (54)	108 (56)	0.9203	70 (53)	98 (58)	0.0007
South	37 (21)	39 (20)		42 (32)	28 (17)	
Midwest	15 (8)	18 (9)		17 (13)	20 (12)	
Northeast	29 (16)	27 (14)		4 (3)	23 (14)	

^#^Unmarried including divorced, separated, single (never married), unmarried or domestic partner, and widowed.

### Impact of chemotherapy on survival in patients with neuroendocrine carcinoma

**Figure 3:**
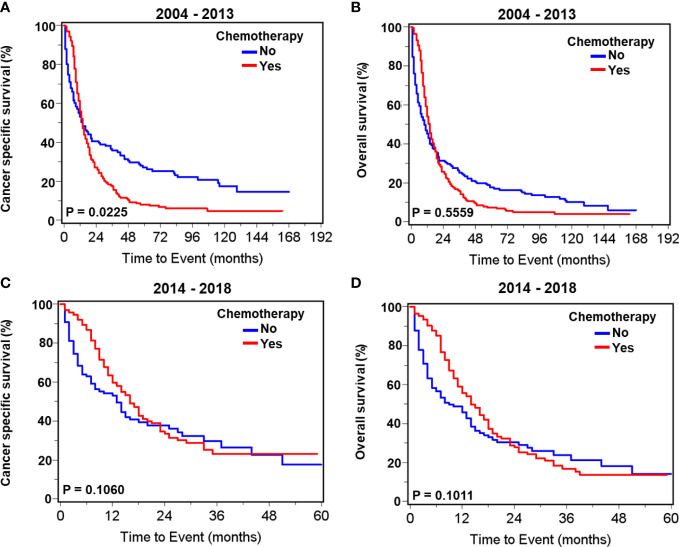
Kaplan–Meier survival curves for cancer-specific and overall survival in patients with *de novo* neuroendocrine prostate carcinoma with or without chemotherapy. For patients diagnosed during 2004–2013, curves of cancer- specific survival **(A)** and overall survival **(B)**. For patients diagnosed during 2014–2018, curves of cancer- specific survival **(C)** and overall survival **(D)**.

Patients with prostate neuroendocrine carcinoma receiving chemotherapy had significantly higher proportions of cancer- specific death and overall death compared to those without chemotherapy during 2004–2013. During 2014–2018, the proportions of cancer- specific and overall deaths were comparable between chemotherapy and no-chemotherapy groups (**Supplementary Table S6**). Survival curves showed that chemotherapy was associated with slightly worse cancer- specific survival (*p*=0.0225) but not overall survival (*p*=0.5559) in patients with neuroendocrine carcinoma during 2004–2013 (**Figures 3A, B**). Chemotherapy was not associated with cancer- specific (*p*=0.1060) and overall (*p*=0.1011) survival during 2014–2018 (**Figures 3C, D**). Multivariate survival analyses showed that chemotherapy was associated with improved cancer- specific survival (HR= 0.62, 95% CI: 0.45–0.87, *p*=0.0055) and overall survival (HR=0.69, 95% CI: 0.51–0.94, *p* = 0.0176) in patients with neuroendocrine carcinoma during 2014–2018. Conversely, chemotherapy was not significantly associated with cancer- specific survival (HR = 0.99, 95% CI: 0.76–1.29, *p* = 0.9138) and survival (HR= 0.89, 95% CI: 0.70–1.14, *p*=0.3540) during 2004–2013 (**Table 7**).

**Table 7:** Multivariate analyses of risk factors related to survival in patients with a *de novo* diagnosis of prostate neuroendocrine carcinoma during 2004–2013 and 2014–2018.

Variable	Cancer- specific survival				Overall survival			
	2004–2013		2014–2018		2004–2013		2014–2018	
	HR (95% CI)	*p*-value	HR (95% CI)	*p*-value	HR (95% CI)	*p*-value	HR (95% CI)	*p*-value
Ag (years)								
≤50	1		1		1		1	
51–60	1.30 (0.70–2.41)	0.4163	1.15 (0.44–3)	0.7763	1.30 (0.69–2.45)	0.4226	0.92 (0.38–2.23)	0.8509
61–70	1.26 (0.68–2.31)	0.4622	1.61 (0.93–2.77)	0.0897	1.25 (0.68–2.33)	0.4724	1.37 (0.62–3.03)	0.4371
71–80	2.11 (1.14–3.90)	0.0181	1.47 (0.83–2.62)	0.1881	2.40 (1.29–4.45)	0.0057	1.42 (0.63–3.20)	0.3983
>80	2.52 (1.34–4.75)	0.0041	2.64 (1.45–4.83)	0.0016	2.89 (1.53–5.44)	0.001	2.52 (1.09–5.84)	0.0313
PSA (ng/ml)								
< 20.0	1				1			
20.0–90.0	0.61 (0.39–0.94)	0.0243			0.64 (0.43–0.95)	0.0274		
>90	1.01 (0.71–1.42)	0.9767			1.04 (0.75–1.45)	0.8021		
Unknown	1.14 (0.80–1.62)	0.4645			1.29 (0.95–1.77)	0.1039		
T stage								
T1	1				1			
T2	0.87 (0.61–1.24)	0.4383			1.41 (0.93–2.14)	0.1029		
T3	0.76 (0.56–1.04)	0.0892			1.32 (0.91–1.91)	0.1381		
T4	0.64 (0.44–0.93)	0.0194			1.73 (1.22–2.47)	0.0023		
N stage								
N0	1				1			
N1	1.36 (1.04–1.76)	0.0226			1.34 (1.04–1.71)	0.0219		
M stage								
M0	1		1		1		1	
M1a	1.54 (0.88–2.71)	0.1342	0.41 (0.15–1.13)	0.0842	1.37 (0.81–2.31)	0.2474	0.48 (0.21–1.11)	0.0851
M1b	1.84 (1.29–2.63)	0.0008	1.16 (0.73–1.85)	0.5334	1.61 (1.15–2.25)	0.0058	1.15 (0.76–1.73)	0.5191
M1c	2.31 (1.68–3.18)	<0.0001	3.88 (2.62–5.75)	<.0001	2.47 (1.84–3.31)	<.0001	3.24 (2.26–4.65)	<0.0001
M1x	1.95 (0.92–4.14)	0.0827	1.32 (0.52–3.36)	0.5613	1.37 (0.81–2.31)	0.2474	1.21 (0.52–2.83)	0.6649
Gleason score								
≤6	1							
7	1.05 (0.35–3.11)	0.9337						
8	0.96 (0.41–2.27)	0.9270						
9–10	1.42 (0.71–2.82)	0.3216						
Unknown	2.04 (1.03–4.01)	0.0399						
Chemotherapy								
No	1		1		1		1	
Yes	0.99 (0.76–1.29)	0.9138	0.62 (0.45–0.87)	0.0055	0.89 (0.70–1.14)	0.3540	0.69 (0.51–0.94)	0.0176
Marital status								
Married			1		1		1	
Unmarried#					1.33 (1.03–1.71)	0.0284	1.38 (1.01–1.89)	0.0419
Unknown					0.80 (0.46–1.38)	0.4164	0.8 (0.37–1.75)	0.5756
Race								
White	1				1			
Black	1.08 (0.70–1.66)	0.7311			0.89 (0.58–1.39)	0.6127		
Other	2.06 (1.09–3.90)	0.0267			1.87 (1.05–3.34)	0.0346		
Unknown	1.72 (0.23–12.94)	0.5976			1.78 (0.23–13.51)	0.5791		
Region								
West					1			
South					1.45 (1.08–1.95)	0.0133		
Midwest					0.94 (0.63–1.42)	0.7784		
Northeast					0.96 (0.69–1.34)	0.8289		

#Unmarried including divorced, separated, single (never married), unmarried or domestic partner, and widowed.

## Discussion

The SEER 18 database, capturing approximately 28% of the total United States population, provides a valuable resource to assess patterns of care for prostate cancer. We specifically examined the utilization of systemic cytotoxic chemotherapy for men with metastatic disease at an initial diagnosis from 2004 to 2018, a period when results of clinical trials suggested new strategies for care. As expected, few men not showing metastatic disease received initial chemotherapy throughout the period. During 2004–2013, the proportion of patients with *de novo* metastatic adenocarcinoma receiving chemotherapy was low (5.8%) but significantly increased to an average of 21.4% during 2014 –2018. The pattern observed likely represents a shared decision- making process (27) between the patient and provider throughout the interval (2004–2018) with utilization of chemotherapy increasingly being offered as an option following publication of new clinical trial results approximately 2014 (10, 11, 22, 23). By 2014, National Comprehensive Cancer Network (NCCN) guidelines recommended ADT plus docetaxel for six cycles as one of several options for the initial treatment of castration- naive metastatic prostate adenocarcinoma (24). Multivariate survival analysis of data from 2004 to 2013 showed that chemotherapy in men with distant metastasis was associated with worse cancer-specific and overall survival; by contrast, it was associated with improved prognosis during 2014–2018.

This is most likely related to patient selection, with chemotherapy being used for men with the most ominous presentation. In contrast to adenocarcinoma, men with neuroendocrine prostate carcinoma (0.1% of all prostate cancer) show an average of 54% of patients receiving chemotherapy with no clear directional change over the entire period. Interestingly, chemotherapy was associated with improved cancer-specific and overall survival in neuroendocrine carcinoma patients during 2014–2018 but not during 2004–2013, perhaps due to improvements in supportive care and patient selection.

For decades, studies of cytotoxic chemotherapy failed to demonstrate benefits for men with metastatic prostate cancer due to challenges in objectively measuring response for a disease dominated by nodal and bone metastasis coupled with a lack of efficacy (28–30). The approval of mitoxantrone for pain control established chemotherapy as an option for men with advanced metastatic disease in 1999 (16, 31). By 2004, a landmark series of studies showed that docetaxel-based therapy for the first time demonstrated improved survival for men with metastatic- castration-resistant prostate adenocarcinoma (17, 18, 31). Newer studies refined our knowledge and documented benefits dependent upon dose intensity (32) and that taxane analogues may prolong benefits (21). Such progress led investigators to consider moving docetaxel chemotherapy into initial treatment strategies for newly diagnosed hormone-sensitive metastatic prostate cancer. Trials showing improved biochemical progression-free survival with the addition of docetaxel to ADT in metastatic hormone-naive prostate cancer patients were emerging by 2013 (23), and by 2015, studies were showing that upfront docetaxel chemotherapy improved overall survival, failure-free survival, and progression-free survival (10, 22, 23). Our study examines how this knowledge impacted therapy for prostate cancer patients in the non-protocol standard practice over the time frame that these results became available (33).

As expected, our study revealed that chemotherapy for those with metastatic adenocarcinoma at diagnosis was sparingly employed at 5.8% prior to 2014 followed by a dramatic rise to nearly 30% from 2014 to 2016. The rapid change was certainly driven by the enthusiasm derived from the new studies and guidelines (23). Although it is possible that the wider insurance coverage becoming available at this time resulting from the American Afford Care Act (34) contributed somewhat, we suspect that the main driving force of adoption was the published research and NCCN guidelines. Yet, it may be surprising to some that only 20%–30% are receiving upfront chemotherapy after 2013–2014. We suspect that both providers and patients contribute to these findings. Our analysis indicates that practitioners are using a range of criteria in patient selection for early chemotherapy, which of course is then modulated by patient preferences after considering risks and benefits. Men receiving initial chemotherapy are younger, typically married (perhaps a marker of support systems), with more advanced T stage, higher Gleason score, positive lymph node metastasis, and more advanced M staging. Thus, it is likely that patients perceived to have a more aggressive phenotype based upon established risk factors are more likely to be offered chemotherapy by practitioners. Of course, we do not have data regarding the initial discussion of options for these men and what percentage were offered chemotherapy and declined. One additional factor may be referral patterns with the urologist typically serving as the initial focal point for diagnosis and perhaps not having medical oncology engaged at the time of the initial treatment plan. Clearly, with only 30% of men receiving chemotherapy, the perceived benefits do not exceed risks in the minds of practitioners and or patients.

Interestingly, we found that from 2004 to 2013, the administration of chemotherapy was associated with worse prognosis compared with no chemotherapy. As chemotherapy became more widespread, we observed a significantly improved cancer- specific and overall survival during 2014–2018. Notably, these findings are verified in subsequent propensity score matching analyses. The poorer outcomes in 2004–2013 likely represents the use of initial chemotherapy for men with greater cancer volume and more aggressive disease, a subgroup destined to have a shorter life expectancy. The lack of a standard of care chemotherapy regimen (agents, dose, and duration) for *de novo* distant metastatic patients may also attribute to the worse outcome during the period. Multiple factors, such as cancer grade, distribution of lesions and volume of disease, PSA, age, and other variables impact offering the chemotherapy option. A previous publication, employing an older version of SEER database (2014–2015) with far less data, also found that chemotherapy-exposed prostate cancer patients exhibited significantly better overall survival (HR=0.82, 95% CI: 0.72–0.96, p=0.01) compared to their chemotherapy- naive counterparts (35). The finding was confirmed in propensity score matching analyses (multivariable HR: 0.77, 95% CI: 0.66-0.90, *p*<0.001) (35). Utilizing a large national cancer database in the United States (2014–2015), another retrospective cohort study revealed that upfront chemotherapy was associated with longer overall survival (HR= 0.78, 95% CI: 68–0.89, *p* < 0.001) in men with *de novo*, treatment- naive metastatic prostate cancer after adjusting for patient and clinical variables (36).

Due to lack of precise data in the SEER database regarding lesion numbers, size, and locations of metastasis, it is not possible to precisely define the high-volume disease. Our multivariate survival analysis suggests that chemotherapy displayed a more beneficial impact for men with potentially higher volume. Men with the nodal metastasis experience no benefit, whereas those with bone metastasis fare better with chemotherapy, and the greatest benefit is seen in men with the visceral disease with or without bone and nodal metastasis.

Not surprisingly, age is shown to be a risk factor for death and particularly strong over the age of 80 years. This and other studies suggest that chemotherapy is less often prescribed for older patients with metastatic prostate cancers (37, 38) and likely due perceived risks associated with frailty, accumulating comorbidities, and poor resilience. Interestingly, we find the benefit of chemotherapy to be best in the ages of 70 –80 years, both on cancer- specific and overall survival. Those younger than 70 and older than 80 tended to gain little or no benefit from chemotherapy. Perhaps, younger men with *de novo* metastatic prostate cancer have a more aggressive disease that is less sensitive to therapy, while older men have comorbidities impacting resilience and tolerability. Similar to our finding, another report suggests that chemotherapy plus ADT, compared to ADT alone, was associated with improved overall survival in *de novo* metastatic prostate cancer patients ≥70 years but not in patients <70 years (39).

Our multivariate survival analyses quantitate the impact of several relevant variables on survival in this cohort. We have limited data on the impact of integrating radiotherapy and surgery but do see a worse outcome noted for those receiving both. It is possible that men with significant and symptomatic local disease have a more aggressive phenotype or medical complications that impact survival. We see a clear trend for married men doing better in survival, perhaps a marker for stronger support systems and compliance with care plans. We did not detect a difference in response based on black vs. white race, but the Southern region of the United States consistently shows poorer survival than the Midwest, with the West and Northeast being similar, perhaps reflecting the impact of social and economic issues on health care access and quality (40, 41). Higher grade cancers and greater disease burden, as indicated by PSA, TNM staging, and Gleason scoring, were strongly related to poor outcomes for those presenting with *de novo* metastatic adenocarcinoma.

Neuroendocrine carcinoma is the rare histological type of prostate cancer with the worst prognosis (25, 42). The disease is typically defined histopathologically and often characterized by lower PSA secretion, higher risk of metastasis, an inferior response to ADT, and poor prognosis (26, 43–45). Small early studies supported the use of chemotherapy with agents often used for cancers of other tissue origins with neuroendocrine features (46). Chemotherapy, radiotherapy, or combinations are associated with improved overall survival compared with palliative therapy (47). We observe that an average of 54% of patients with neuroendocrine prostate cancer are treated with first-line chemotherapy and is steady from 2004 to 2018. Chemotherapy is associated with improved cancer-specific and overall survival during 2014–2018, but not during 2004–2013, perhaps due to improved supportive care plans and better patient selection. Hence, our findings may support the use of chemotherapy for both *de novo* and treatment-emergent neuroendocrine prostate cancer due to the potential survival benefits.

This retrospective study has limitations. Our study is subject to the constraints of the SEER database, including the precision of data collection and the number of variables collected. There is a lack of information on the specific chemotherapy regime, dose of drug, compliance, and dose intensity including the number of cycles. The database has no information on concurrent ADT and the types or duration of agents provided. Indeed, we suspect that the increased use of effective agents impacting androgen receptor signaling may reduce the frequency of selecting taxane-based chemotherapy in the up-front approach to *de novo* metastatic disease since 2016 (**Figure 1**). There is no information on other important outcomes such as toxicities of chemotherapy, quality of life, recurrence of cancer and additional therapy, and the dynamic change in PSA. Our study is limited by the inherent challenges of a retrospective cohort design. For example, it is likely that patients with better performance status were selected for chemotherapy, which may contribute to better survival outcomes. Hence, selection bias is inevitable but is clearly a component of practice decisions. The strength of this study is the very large sample size providing accurate insight into practice patterns in a real world setting during a period when new data were emerging.

## Conclusions

Chemotherapy has been increasingly employed in the community for men with *de novo* metastatic adenocarcinoma at diagnosis following a series of publications in 2013–2014, yet for less than one-third of men. Our data suggest that both medical practitioners and patients may be carefully considering the risks and benefits for each individual based upon age, histopathological features, PSA, staging criteria, comorbidities, and a number of factors such as overall performance status. Findings of this study support the initial treatment with chemotherapy in men in the 70–80 age group presenting *de novo* with more aggressive features or greater volume of metastatic disease. Clearly, our work suggests that shared decision making is the strategy in the community for men presenting with metastatic adenocarcinoma who are mostly seniors and often with comorbidities. In contrast, the treatment of neuroendocrine prostate cancer with initial chemotherapy has been stable at approximately 50%, but with improving outcomes in recent years.

## Data availability statement

The original contributions presented in the study are included in the article/**Supplementary Material**. Further inquiries can be directed to the corresponding authors.

## Ethics statement

Ethical review and approval was not required for the study on human participants in accordance with the local legislation and institutional requirements. Written informed consent for participation was not required for this study in accordance with the national legislation and the institutional requirements.

## Author contributions

Concept and design: SW and SC. Data acquisition and analysis: SW and SC. Interpretation of data: all authors. Drafting of the manuscript: SW and SC. Critical revision of the manuscript for important intellectual content: all authors. Supervision: SC. All authors contributed to the article and approved the submitted version.
