# Supplementary material for: Chemotherapy for the initial treatment of metastatic prostate adenocarcinoma and neuroendocrine carcinoma at diagnosis: real world application and impact in the SEER database (2004 –2018)
Source: Front Oncol. 2023 Jun 9;13:1165188. doi: 10.3389/fonc.2023.1165188 (PMC10288985; doi:10.3389/fonc.2023.1165188)

Supplemental data

Table S1. Descriptive demographic and clinicopathological characteristics of patients with prostate adenocarcinoma or neuroendocrine carcinoma diagnosed during 2004-2018.

**Variable**	**Adenocarcinoma n (%)**	**Neuroendocrine carcinoma n (%)**	**P value**
	**727,133 (99.9)**	**671 (0.1)**	
**Age (years)**			
Mean ± SD	66.2 ± 9.1	70.1 ± 11.1	<0.0001
Median (range)	66 (20-100)	70 (30-96)	
Distribution			<0.0001
≤50	27,565 (4)	27 (4)	
51-60	171,181 (24)	100 (15)	
61-70	302,567 (42)	228 (34)	
71-80	180,265 (25)	186 (28)	
>80	45,555 (6)	130 (19)	
**PSA (ng/ml)**			<0.0001
Mean ± SD	15.3 ± 24.0	29.3 ± 37.6	
Median (range)	6.8 (0.1-99.8)	8.5 (0.1-99.8)	
Distribution			<0.0001
< 20	566,067 (78)	381 (57)	
20-90	54,078 (7)	81 (12)	
>90	41,542 (6)	111 (17)	
Unknown	65,446 (9)	98 (15)	
**T stage**			<0.0001
T1	566,067 (78)	381 (57)	
T2	54,078 (7)	81 (12)	
T3	41,542 (6)	111 (17)	
T4	65,446 (9)	98 (15)	
**N stage**			<0.0001

N0	705,781 (97)	354 (53)	
N1	21,352 (3)	317 (47)	
**M stage**			<0.0001
M0	703,129 (97)	288 (43)	
M1a	1,689 (0)	39 (6)	
M1b	17,014 (2)	125 (19)	
M1c	4,110 (1)	201 (30)	
M1x	1,191 (0)	18 (3)	
**Gleason score**			<0.0001
≤6	291,203 (40)	20 (3)	
7	273,233 (38)	26 (4)	
8	66,059 (9)	37 (6)	
9-10	57,004 (8)	191 (28)	
Unknown	39,634 (5)	397 (59)	
**Local treatment**			<0.0001
No	196,020 (27)	277 (41)	
Radiotherapy only	230,786 (32)	160 (24)	
Surgery only	278,317 (38)	165 (25)	
Radiotherapy and surgery	22,010 (3)	69 (10)	
**Chemotherapy**			<0.0001
No	721,584 (99)	310 (46)	
Yes	5549 (1)	361 (54)	
**Marital status**			<0.0001
Married	479,050 (66)	457 (68)	
Unmarried#	156,902 (22)	182 (27)	
Unknown	91,181 (13)	32 (5)	
**Race**			<0.0001
White	566,736 (78)	569 (85)	
Black	110,193 (15)	61 (9)	
Other	36,919 (5)	38 (6)	

Unknown	13,285 (2)	3 (0)	
**Region**			0.0001
West	356,396 (49)	372 (55)	
South	170,918 (24)	146 (22)	
Midwest	73,425 (10)	70 (10)	
Northeast	126,394 (17)	83 (12)	

Unmarried including divorced, separated, single (never married), unmarried or domestic Partner, widowed.

Table S2. Cancer specific or overall death in patients with a *de novo* diagnosis of metastatic prostate adenocarcinoma who were initially treated with or without chemotherapy during 2004-2013 and 2014-2018.

	**2004-2013**			**2014-2018**		
	**No chemotherapy 12,451 (94%)**	**Chemotherapy 772 (6%)**	**P value**	**No chemotherapy 8,471 (79%)**	**Chemotherapy 2,310 (21)**	**P value**
**Follow-up time (months)**						
**Mean ± SD**	100.0 ± 42.3	74.4 ± 50.9	<0.0001	26.4 ± 17.3	23.1 ± 14.6	<0.0001
**Median (range)**	100 (0-179)	70 (0-179)		25 (0-59)	22 (0-59)	
**Overall death (%)**	83	91	<0.0001	37	32	<0.0001
**Cancer specific death (%)**	64	80	<0.0001	29	28	0.3654
**Proportion of overall death due to prostate cancer (%)**	77	88	<0.0001	79	89	<0.0001

Table S3. Descriptive characteristics of propensity score matched patients with a *de novo* diagnosis of metastatic prostate adenocarcinoma who were initially treated with or without chemotherapy during 2004-2013 and 2014-2018.

	**2003-2014 n (%)**			**2014-2018 n (%)**		
**Variable**	**No Chemotherapy 772 (50)**	**Chemotherapy 772 (50)**	**P value**	**No Chemotherapy 2,310 (50)**	**Chemotherapy 2,310 (50)**	**P value**
**Age (years)**						
Distribution			0.9997			0.2116
≤50	77 (7)	77 (7)		101 (4)	134 (6)	
51-60	302 (28)	302 (28)		572 (25)	564 (24)	
61-70	466 (43)	462 (42)		1031 (45)	990 (43)	
71-80	197 (18)	200 (18)		506 (22)	515 (22)	
>80	47 (4)	48 (4)		100 (4)	107 (5)	
**PSA (ng/ml)**				101 (4)	134 (6)	
Distribution			0.8779			0.5733
<20.0	612 (56)	624 (57)		448 (19)	448 (19)	
20-90.0	285 (26)	275 (25)		669 (29)	628 (27)	
>90	128 (12)	121 (11)		1138 (49)	1178 (51)	
Unknown	64 (6)	69 (6)		55 (2)	56 (2)	
**Gleason score**			0.7152			0.4620
≤6	43 (4)	51 (5)		22 (1)	23 (1)	

7	227 (21)	226 (21)		172 (7)	172 (7)	
8	278 (26)	268 (25)		419 (18)	415 (18)	
9-10	485 (45)	476 (44)		1,451 (63)	1,425 (62)	
Unknown	56 (5)	68 (6)		246 (11)	275 (12)	
**T stage**			0.9296			0.8102
T1	212 (19)	224 (21)		759 (33)	728 (32)	
T2	347 (32)	346 (32)		762 (33)	779 (34)	
T3	423 (39)	415 (38)		369 (16)	374 (16)	
T4	107 (10)	104 (10)		420 (18)	429 (19)	
**N stage**			0.3597			0.1260
N0	883 (81)	866 (80)		1,197 (52)	1,145 (50)	
N1	206 (19)	223 (20)		1,113 (48)	1,165 (50)	
**M stage**			0.9824			0.5316
M1a	50 (6)	53 (7)		131 (6)	126 (5)	
M1b	482 (62)	480 (62)		1641 (71)	1607 (70)	
M1c	205 (27)	202 (26)		405 (18)	425 (18)	
M1x	35 (5)	37 (5)		133 (6)	152 (7)	
**Marital status**			0.7705			0.9593
Married	718 (66)	731 (67)		1,433 (62)	1,442 (62)	
Unmarried[#]	278 (26)	273 (25)		741 (32)	732 (32)	
Unknown	93 (9)	85 (8)		136 (6)	136 (6)	
**Race**			0.9824			0.7234

White	885 (81)	887 (81)		1,798 (78)	1,769 (77)	
Black	146 (13)	141 (13)		367 (16)	387 (17)	
Other	55 (5)	58 (5)		136 (6)	142 (6)	
Unknown	3 (0)	3 (0)		9 (0)	12 (1)	
**Region**			0.8757			0.4050
West	617 (57)	618 (57)		1235 (53)	1188 (51)	
South	197 (18)	204 (19)		557 (24)	558 (24)	
Midwest	118 (11)	107 (10)		207 (9)	229 (10)	
Northeast	157 (14)	160 (15)		311 (13)	335 (15)	
**Local treatment**			0.8878			0.8989
Radiotherapy only	176 (16)	172 (16)		1,616 (70)	1,613 (70)	
Surgery only	346 (32)	335 (31)		479 (21)	468 (20)	
Radiotherapy and surgery	394 (36)	397 (36)		166 (7)	178 (8)	
Radiotherapy only	173 (16)	185 (17)		49 (2)	51 (2)	

Unmarried including divorced, separated, single (never married), unmarried or domestic Partner, widowed.

Table S4. Cancer specific or overall death in propensity score matched patients with a *de novo* diagnosis of metastatic prostate adenocarcinoma who were initially treated with or without chemotherapy during 2004-2013 and 2014-2018.

	2004-2013			2014-2018		
**Chemotherapy**	**No 772 (50%)**	**Yes 772 (50%)**	**P value**	**No 2,310 (50%)**	**Yes 2,310 (50%)**	**P value**
**Overall death (%)**	81	91	<0.0001	37	32	0.0003
**Cancer specific death (%)**	67	80	<0.0001	30	28	0.1548
**Proportion of overall death due to prostate cancer (%)**	83	88	0.0149	82	89	<0.0001

Table S5.

Treatments received in patients with prostate neuroendocrine carcinoma during 2004-2018.

Variable	Chemotherapy		P value
	**No n(%)**	**Yes (n%)**	
	**310 (46)**	**361 (54)**	
**Local treatment**			<0.0001
**No local treatment**	142 (46)	135 (37)	
**Radiotherapy only**	42 (14)	118 (33)	
**Surgery only**	103 (33)	62 (17)	
**Radiotherapy and surgery**	23 (7)	46 (13)	

Table S6. Cancer specific or overall death of patients with a *de novo* diagnosis of prostate neuroendocrine carcinoma who were initially treated with or without chemotherapy during 2004-2013 and 2014-2018.

	**2004-2013**			**2014-2018**		
**Chemotherapy (n/%)**	**No 177 (48%)**	**Yes 192 (52%)**	**P value**	**No 133 (44%)**	**Yes 169 (56%)**	**P value**
**Follow-up time (months)**						
**Mean ± SD**	27 ± 39.1	20.8 ± 24.1	<0.7857	10.9 ± 13.5	13.9 ± 11.6	0.0005
**Median (range)**	9 (0-167)	13 (0-162)		6 (0-59)	11 (0-58)	
**Overall death (%)**	88	95	0.0213	66	68	0.7294
**Cancer specific death (%)**	68	90	<0.0001	56	56	0.9206
**Proportion of overall death due to prostate cancer (%)**	77	95	<0.0001	84	83	0.0785

Figure S1.

Kaplan-Meier survival curves for cancer specific and overall survival in propensity score matched patients with *de novo* metastatic prostate adenocarcinoma with or without chemotherapy. For patients diagnosed during 2004-2013, curves of cancer specific survival (A) and overall survival (B). For patients diagnosed during 2014-2018, curves of cancer specific survival (C) and overall survival (D).

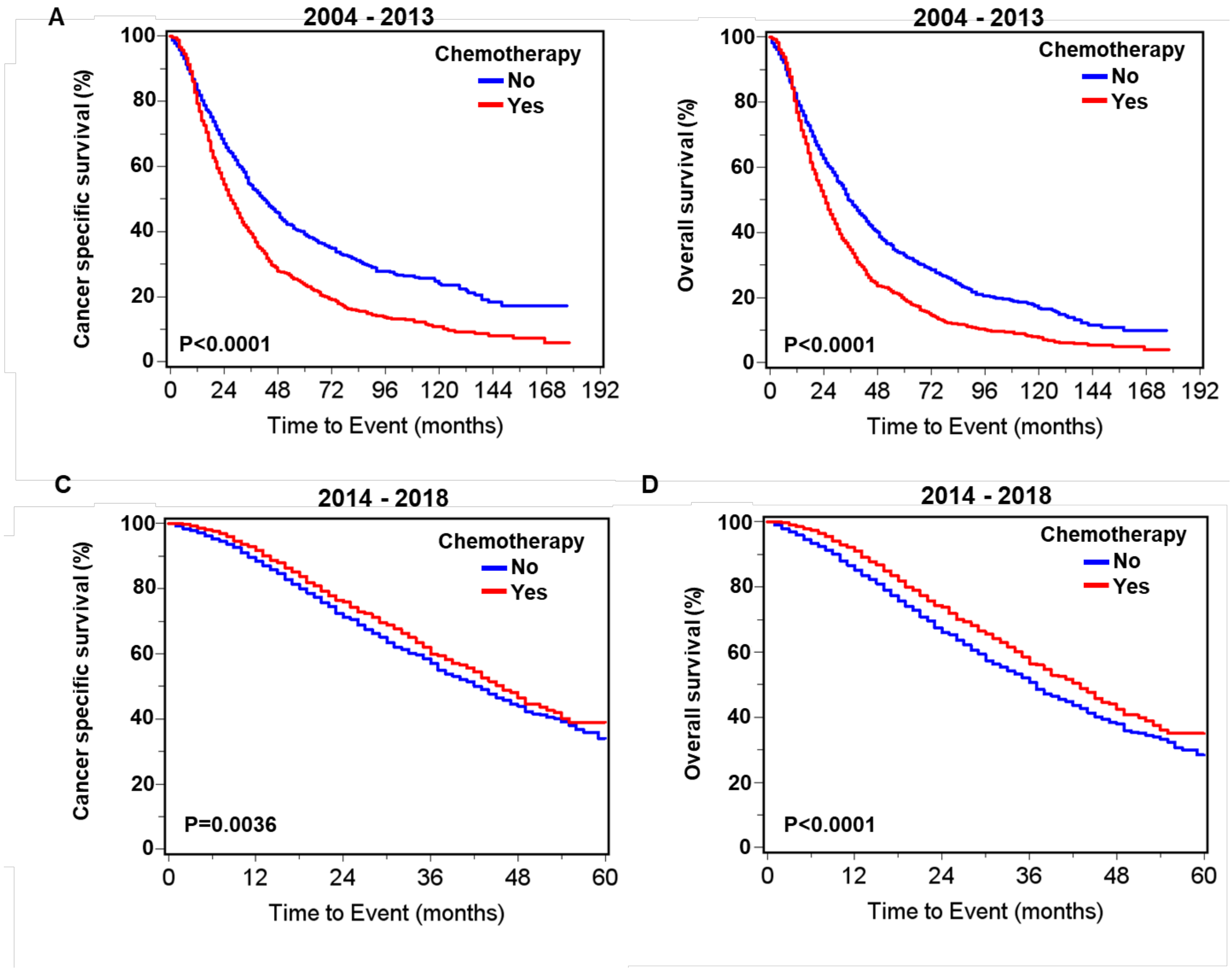

Supplement: Supplementary file 1 [file DataSheet_1.pdf]
